# Assessment of the medical equipment supply chain in the Democratic Republic of Congo: a qualitative methods study

**DOI:** 10.1186/s12913-026-14131-y

**Published:** 2026-02-05

**Authors:** Jonathan Niles, Marc Bosonkie, Paul-Samson Lusamba-Dikassa, Janna Wisniewski

**Affiliations:** 1 Department of International Health and Sustainable Development, Celia Scott Weatherhead School of Public Health and Tropical Medicine, Tulane University, New Orleans, USA; 2 School of Public Health, University of Kinshasa, Kinshasa, Democratic Republic of the Congo

**Keywords:** Democratic Republic of Congo, Medical equipment, Supply chain, Health information systems, Qualitative methods

## Abstract

**Background:**

Efforts to improve health-related supply chains in the Democratic Republic of Congo (DRC) have typically focused on medicines and vaccines, while medical equipment has often been either overlooked or considered incidental. While national guidance exists on the procurement, maintenance, and reporting on medical equipment, the extent to which these norms are implemented in practice is unclear. This study qualitatively assessed the design and operational functioning of the medical equipment system in the DRC, with a focus on information systems, procurement processes, maintenance, and depreciation, and examined how roles and responsibilities shape equipment availability and functionality at health facilities.

**Methods:**

A qualitative research design combined a desk review of national policies and in-depth interviews with key stakeholders at health facilities, health zone offices, provincial health departments, the Ministry of Health, and developmental partner organizations in the DRC. Interviews were analyzed using a hybrid approach of content and thematic analysis.

**Results:**

Findings revealed several weaknesses in the medical equipment system in the DRC. Many health facilities, particularly health centers, lacked the minimum required equipment, which adversely affected the quality of services provided. Responsibility for equipment procurement and maintenance was not clearly defined, leading to a lack of accountability and coordination. We identified a disconnect between data availability, resource mobilization, and decision making within the health system. Both equipment procurement and maintenance were governed by unclear and fragmented processes.

**Conclusions:**

Overall, the medical equipment system in the DRC is not designed to meet population needs, and in practice the system is not functioning as designed, which contributes to inadequate availability and maintenance of equipment in health facilities. Based on the barriers identified, recommendations include establishing a formal process to keep facilities informed of equipment requests as well as exploring regular planning and budgeting exercises for equipment procurement. The potential benefits of subsidies for equipment purchases, in certain cases, are also highlighted. The findings from this study offer a blueprint for assessing medical equipment systems in low-income countries, highlighting the importance of this often-overlooked component of quality health services.

**Supplementary Information:**

The online version contains supplementary material available at 10.1186/s12913-026-14131-y.

## Background

Medical equipment and supplies are essential for health service delivery. According to the World Health Organization (WHO), a well-functioning health system should “ensure equitable access to essential medical products, vaccines and technologies of assured quality, safety, efficacy and cost-effectiveness, and their scientifically sound and cost-effective use” [1]. Yet, the adoption of many health technologies fails in practice due to a lack of investment to introduce, maintain, and manage these technologies sustainably [2]. A well-functioning medical equipment system is often a complex relationship between manufacturers who source individual components from networks of suppliers, with finished goods offered to group purchasing organizations, governments, distributors, or individual customers, which may include hospitals, pharmacies, physicians, or patients. Many medical devices require maintenance and repair which may involve additional components, services, and providers [3]. The equipment shortages observed during the COVID-19 pandemic have renewed global attention to the resilience and governance of national medical equipment systems [4], leading to the creation of national databases to monitor equipment supply chains and shortages [5].

As is the case in many low-income countries, efforts to improve health-related supply chains in the Democratic Republic of Congo (DRC) have tended to focus on medicines and vaccines. Supply chains in the DRC are siloed, with commodities such as essential medications, vaccines, bed nets, and contraceptive devices distributed through parallel systems operated by different health programs. Assessments of the medical equipment system suggest that it is fragmented in funding sources, procurement processes, and administration [6–8]. Responsibility for equipping health facilities and performing maintenance on existing equipment appears to rest at multiple levels, with limited accountability throughout. While medicine stocks and service delivery data are reported in the routine health information system, there does not appear to be a centralized system for detailed tracking of medical equipment.

**Fig. 1:**
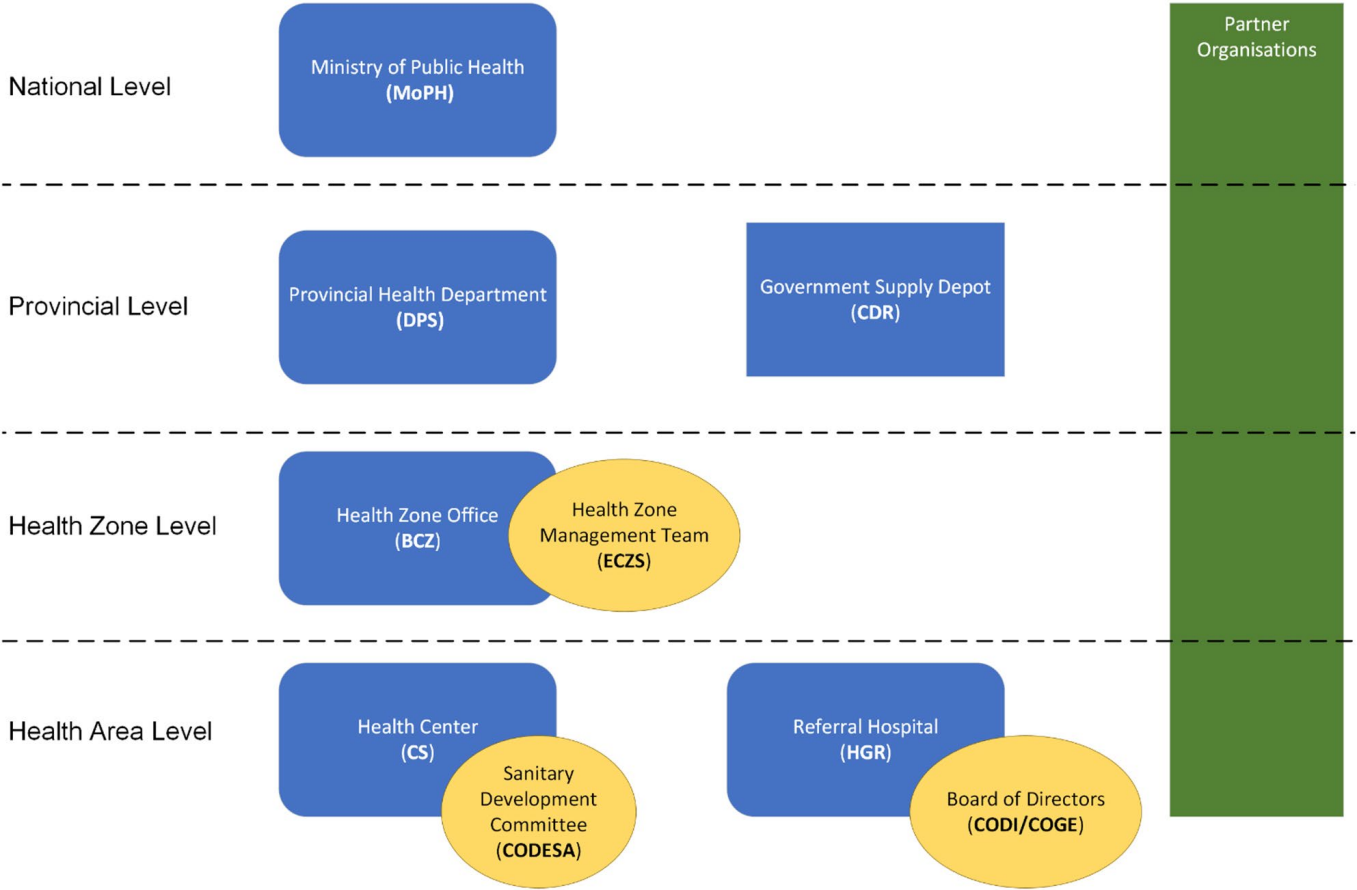
Levels of health delivery in the Democratic Republic of Congo

The DRC’s health system is decentralized and multileveled, and operates across provincial health departments, health zones, and health areas as shown in Fig. 1. The Ministry of Health (MoH) sets health policies and standards, while provincial health departments (*Direction Provinciale de la Santé* [DPS]) ensure healthcare delivery. Health zones, managed by the health zone office (*Bureau Central de la Zone de Santé* [BCZ]) and management team (*Equipe Cadre de la Zone de Santé* [ECZ]) include a referral hospital (HGR) and health centers (CS), the latter overseen by a head nurse in collaboration with the Sanitary Development Committee (CODESA). The health area and health zone levels report health data through the routine health information system (*Système National d’Information Sanitaire* [SNIS]).

A substantial portion of the health delivery is provided by international governments and non-governmental organizations [8]. Partner organizations of the Ministry of Public Health are involved at all levels of the health system, contribute financial and technical support to health services. National religious organizations are also an important governmental partner in health service delivery [9]. Private religious health facilities may be nominated as “designated” facilities by the Ministry of Health and are held to the same standards as governmental facilities.

The absence of a well-defined equipment supply chain has a significant impact. In 2014, the evaluation of the *Accès aux Soins de Santé Primaires* (ASSP) project found fewer than 12% of sampled health facilities had adequate equipment (Tulane University School of Public Health and Tropical Medicine, 2019). Mirroring this finding, as part of a performance evaluation of the United States Agency for International Development (USAID) Integrated Health Program (IHP), Data for Impact (D4I) found that only 48.4% of 281 health centers assessed met the national standards for basic equipment in 2021. Hospitals were better equipped in general, although only 79.2% had a full complement of basic equipment in 2021. Furthermore, no significant changes in hospital-based equipment were found between 2019 and 2021 [10].

The government of the DRC has recognized the importance of improving the medical equipment landscape. The National Plan for Health Development for 2019–2022 prioritized “strengthening the maintenance of infrastructure and equipment acquired.” Specifically, this directive stated that “standards relating to the management of equipment and materials will be updated and disseminated. … A policy of depreciation and regular maintenance of Ministry of Public Health equipment, materials, and infrastructure at all levels will be instituted. Awarding contracts with centers of expertise will be considered, as well as a capacity building plan for the maintenance centers of the Ministry of Public Health” [9]. This development goal emphasizes the need for an evaluation of how the medical equipment supply chain has been functioning to inform the barriers and facilitators to equipment procurement and maintenance.

There are few published in-depth assessments of medical equipment systems in low-income countries, despite being an important component of quality of care. This study can highlight this often-overlooked system and be used as a template for other countries when assessing their own medical equipment systems. The purpose of this study was to qualitatively assess the design and operational functioning of the medical equipment system in the DRC, with a focus on information systems, procurement processes, maintenance, and depreciation, and to examine how roles and responsibilities, including those of government actors and development partners, shape equipment availability and functionality at health facilities.

## Methods

To meet the study objective, a qualitative research design was used combining a desk review of national policy documents and semi-structured interviews. The desk review examined national policies, procedures, and standard operating procedures related to the medical equipment system obtained through the Ministry of Health website, direct outreach, and key informant interviews. This study took place in the context of a broader health systems evaluation of the USAID IHP.

We identified key stakeholders as the MoH, provincial health departments (DPS), non-governmental organizations (NGOs), health zone offices (BCZs), general reference hospitals (HGRs), and health centers (CSs). Participants were recruited to the study through direct outreach by telephone, obtained from lists provided by the MoH and professional organizations. All participants consented to be interviewed. While the sampling did not consider saturation, prior research suggests that the sample size was sufficient to reach thematic and meaning saturation among health center respondents [11]. The sample sizes and characteristics of each strata are described in Table 1.

Interview guides were developed for key informant interviews (Additional file 4, Additional file 5). The interview guides were pretested at three health facilities in Kinshasa. Key informant interviews were conducted by trained data collectors from the Kinshasa School of Public Health for three provinces (Haut Katanga, Kasaï Oriental, and Tanganyika) and in the capital city Kinshasa from June to July 2022. At a national level, interviews were conducted with officials from the Ministry of Public Health, an official working in equipment procurement. At a provincial level, interviews were conducted with informants from the provincial health department, health centers, hospitals, and health zone offices obtained by purposeful sampling. After initial data collection and analysis, additional interviews were conducted with representatives from international partner organizations of the MoH identified via snowball sampling (*n* = 3) between May 2023 and July 2023. Participants were only interviewed once. All interviews, except two conducted in English, were carried out in French or Lingala and lasted up to one hour.

**Table 1:** Characteristics of qualitative informants

**National Level**		**Total Informants**
MoH Representatives		2
Government Procurement Official		1
INGO Representatives		3
**Provincial Level**	**Sampling**	**Total Informants**
Provincial Equipment Managers		4
Health Zone Chief Physician	Two health zones	6
Head of General Reference Hospital	Two health zones	6
Head Nurse of Health Center	Four health centers	24
**Total**		**42**

In interviews, we asked these informants to validate what is stated in national and provincial policies and provide insight into any discrepancies or gaps in information concerning the medical equipment system’s design and operation. We asked health facility staff and leadership how equipment is procured, tracked, and maintained. Further, we asked about equipment gaps and explored reasons behind why the facility may not have been not adequately equipped. We also discussed whether and how donor support impacts equipment procurement and maintenance. In interviews with international partner organizations, we asked about their experiences procuring equipment, sources of data which informed purchasing decisions, and the organization’s role in the equipment supply chain.

Interviews were audio recorded and transcribed. Transcriptions were not returned to participants due to limitations in access. Data were coded using ATLAS.ti software and subsequently analyzed inductively and deductively by JN, MB, and JW. Based on the interviews and review of policy documents, we mapped the design and functioning of the DRC’s medical equipment system using a hybrid approach of content and thematic analysis.

## Results

The findings presented below address the study objective by describing the design and operational functioning of the medical equipment system in the DRC, with attention to information systems, procurement processes, maintenance and depreciation practices, and the roles and responsibilities of government actors and development partners. The analysis revealed misalignment in equipment data generation, usage, and reporting procedures; unclear responsibility for equipment procurement; and limited promulgation of standards for equipment maintenance and depreciation.

### Demographic profiles of study participants

At the provincial level, most study participants were male (87%), with an average tenure of 7.6 years in their current position. All but two respondents held at least a bachelor’s degree. Among them, 24% possessed a Doctor of Medicine degree, and 69% held a degree in nursing sciences. The remaining participants had degrees in pharmaceutical management, logistics, or commercial sciences.

### Equipment data in the SNIS

#### Data generation and reporting

The desk review revealed a limited set of medical equipment data elements routinely collected by health facilities. According to national policy documents, the mechanism for reporting inoperable medical equipment is embedded within the SNIS, which operates at the level of health center (peripheral level), general reference hospital (health district hospital), health zone office and provincial health office. At each level, data is mandated to be compiled monthly and forwarded to the next level upwards using SNIS data collection forms. In general, health facilities record data on paper-based forms, which are digitized at the health zone office using the District Health Information System 2 (DHIS2) software. DPS’s supervise general reference hospitals, and BCZ’s supervise health centers.

At the health zone office, national standards name an administrator-manager (AG) to oversee the equipment of the zone, both assets and medicines. The AG reports to the chief physician of the zone (MCZ) who in turn oversees a team of nurses who function as zone supervisors. The zone team is responsible for receiving the SNIS reports from institutions in the zone and encoding them into the online SNIS platform.

Both health centers and general reference hospitals report inoperable equipment by specifying the number of days each equipment item was inoperable during the previous month. The equipment list consists of six items: electricity, refrigerator, microscope, glucometer, spectrometer, and centrifuge (Additional file 2). General reference hospitals also report on twelve additional pieces of equipment: computer, photocopier, motorcycle, vehicle, internet, incubators for pre-term and full-term neonates (*incubateur et couveuse*), electrophoresis chain, resuscitation device, echograph, radiograph, and electrocardiogram (Additional file 3).

Once data is uploaded to the DHIS2 server, it is available to provincial and national authorities, including the DPS. Users at each level (health zone, provincial, national) can create custom dashboards featuring data collected in the SNIS.

#### Critiques of equipment data in the SNIS

Informants from both the provincial and national Ministry of Public Health reported relying on the national health information system for access to equipment information. However, they expressed frustration that the data was incomplete and did not reflect the reality found in the health facilities.

The [SNIS] report concerns the minimum package of activities for each facility. … But the reality on the ground is different, for example you go to a health center, you find that they have an operating table, whereas according to the standards of the Ministry of Public Health, one cannot perform an operation in a health center. You find there is an operating table and people are operating there. (Ministry of Public Health)

Informants at all levels of the health system observed that the data in the SNIS was limited.

…there is almost no data that we collect in relation to medical equipment. The few limited data that are in the SNIS form, because the SNIS form does not include the entire list of medical equipment necessary for either the hospital or the health center. (BCZ – Kasaï-Oriental)

… [The SNIS] asks the equipment quantity and the number (of days) of non-functionality. These are the only two pieces of information sought in the SNIS. But perhaps considering the age of the equipment, obsolescence of the equipment, […] this information is no longer there. (Hospital – Kasaï Oriental)

Well, the SNIS framework does not list all the medical equipment, nevertheless there are a few items of equipment that have been requested to always be filled out in the SNIS. But there is not a detailed report of the equipment in the monthly report. […] it only asks how many days the fridge worked, the electricity, the manual centrifuge, microscope. It is only that bit of equipment that gets entered online in the monthly report. (Health center – Kasaï Oriental)

### Parallel equipment tracking system

#### Generation and reporting

Health centers reported tracking the use of medical equipment, the status, the origin (purchased with the institution’s own funds, allocation from the state, nongovernment organization or charitable partner), and the acquisition year outside of the SNIS. Most Informants cited the use of a *Fiche d’Inventaire* (Inventory Sheet) issued by the health zone, though some health centers produced their own registries using pen and paper. These reports are made in duplicate or triplicate, with one copy remaining in the archives of the health center and the others sent to the health zone office and partners.

The information [we track] is about the quantity, the condition, even the origin of the material. This is what is asked [of us]. Even the donation year of the material. So, who provided the equipment, is it a partner, is it the State, is it you yourself who bought on the market? It is those fields that are included. (Health center – Kasaï Oriental).

Hospitals generally record information about their equipment in an inventory report. Each department produces a report for management, and management compiles all the information and sends it to the BCZ when the latter requests it (often quarterly).

No, as soon as we report, as it is each services’ inventory, the information or report is immediately used by the service, to be able to see what is there. So, if there is something to fix, to improve, if there is a piece of equipment that is declared out of use, we remove it, we may put it in the depot and we can make another requisition so that we can replace it. (Hospital – Kasaï-Oriental)

In our policies, it is only if the central office asks, and in that case, we can easily give them [the inventory], but the frequency really is perhaps quarterly, not even monthly. (Hospital – Tanganyika)

Of the hospitals visited, all used paper-based inventory systems as their primary means of data collection. Some HGRs additionally transcribe equipment data into a computerized system.

#### Availability and use

Many health center informants described the open sharing of data on medical equipment among staff and the local community, represented by the president and members of the health development community (CODESA). Informants view the community as a partner in management of the health facility, both as a facilitator and auditor. Community participation is formalized in a few of the health centers by the explicit inclusion of the CODESA during equipment inventory, as well as providing copies of inventory reports sent to the health zone office to the CODESA president.

I told you we have data with supporting documentation. (1) It is found at the [health] center. (2) It is [found] in the community with the president of CODESA and (3) It is at the central office. (Health center – Kasaï Oriental).

No hospital informant mentioned the participation of the community in hospital administrative activities. Rather, data are presented and analyzed during internal staff meetings. In most cases, the hospital administrator collects and archives equipment data but may also share the role with the director of nursing.

Informants reported that equipment reports were available at the health zone office, citing as users of the data the Expanded Program on Immunization (PEV), visiting dignitaries, partner organizations, and the DPS. Access to data often requires authorization from the DPS, but once obtained, is straightforward.

Anyone who needs to know more about the data has the access to enter, to ask questions to the person who keeps it, he brandishes the [authorization] document and finally he can easily access the given data. (Health zone – Tanganyika)

Informants from partner organizations reported performing their own needs assessments prior to implementing programs which required medical equipment purchases. Respondents mentioned bespoke surveys to inform procurement decisions, but none mentioned using the SNIS information system as part of a medical equipment assessment. Two informants reported creating software tools to track equipment to fill their perceived gap in medical equipment tracking.

We discuss with the various partners of the State and the government, and we go to the field to also discuss with the province. Based on this initial assessment, [we] put together a team to write the project [.] considering the real needs of the population in the field. (Implementing Partner).

We created an inventory at the start of the project in 2019 to identify the needs for materials and equipment at the level of the health facilities in which we support. (Implementing Partner).

### Equipment procurement

The designation of the health facility (public, private, non-governmental organization) determines how medical equipment is provided or replaced, as is the source of funding (government, private, bilateral, or multilateral assistance). If public funds are used, health facilities are expected to follow government procedures for acquiring or replacing equipment. These procedures are not specific to the health sector but are standard for all government sectors and services. They are described in official documents (Additional file 1, documents 2–6.)

#### Public funds

According to our desk review of national policies, health facilities may order equipment from government supply depots (*Centre de Distribution Régionales* [CDR]). CDRs are public, not-for-profit institutions, supplying health facilities with medicines, equipment and supplies through direct payment or payment through credit lines. CDRs follow the guidelines and procedures issued by the National Program for the Provision of Essential Medicines (*Programme National d’Approvisionnement en Médicaments Essentiels* [PNAME]) (Additional file 1, document 9).

CDRs are expected to maintain a database of all the health zones in their service area and their respective BCZs. To place an order, a health facility should fill out a procurement form (*bon de commande*) indicating the name, specifications, and quantities of the requested item, and forward it to the BCZ. The BCZ checks the order for conformity to guidelines and checks the credit balance of the health facility before forwarding it to the CDR. At the CDR, similar checks are performed, as well as a verification whether the facility qualifies for service by that CDR. If so, the order is executed, and delivery is organized through the CDR’s distribution system, or the order is picked up by the BCZ or health facility.

When asked about the role of the CDR, most facility-based and BCZ-level informants stated that the CDRs only supplied medicines and did not regularly provide medical equipment. The informant from the CDR in Kinshasa corroborated this, explaining that, while they theoretically could order medical equipment, they did not have the processes nor the budget to do so at the present time.

There was consensus among informants that health facilities are authorized to directly purchase less expensive equipment such as thermometers and blood pressure cuffs. Health centers had annual spending limits for equipment; the reported amount of the spending limit varied, ranging from 10,000 to 100,000 Congolese Francs, and in some cases, 20% of revenue. While many of the informants at the health centers reported that they could purchase equipment directly, for a subset, this authorization was theoretical, as they reported having inadequate funds to purchase any equipment. In at least one health center, the informant did not appear to have considered that the health center could purchase equipment itself, and another informant reported having spent two years trying to raise funds for a purchase.

When a health center directly purchases equipment, the process for informing the BCZ varies. Some informants felt that in an emergency they could buy equipment immediately, but for routine purchases, they are supposed to seek authorization from the BCZ. Other health centers reported their equipment acquisitions to the BCZ afterward. One health center reported that large purchases require authorization from the BCZ, even when the health center is using its own funds.

Hospital informants reported that they also directly purchase equipment when able, with more expensive equipment requested from the hospital’s senior leadership team (*Comité de Direction* [CODI]) or the BCZ. Hospitals tended to make purchasing decisions via management committees. Informants mentioned that while they may work with partners, they do not feel obligated to wait for a partner to bring them a piece of equipment, particularly when the hospital and partner’s priorities may not align.

Some hospitals and health centers create annual operational action plans and six-month management plans, which include new equipment acquisition. The funds for these acquisitions are supposed to be included in the facility’s operating budget. Privately-run facilities tend to be more agile in purchasing equipment directly, as they can draw additional support from the facility’s owner or sponsoring religious institution. In a few instances, informants mentioned that if a health worker or patient breaks a piece of equipment, they are expected to pay for its repair or replacement.

Health facilities tend to buy medical equipment on local markets, although in one case the health center manager had traveled to Dubai and brought equipment back with him. One facility manager explained that they have ongoing relationships with suppliers in the nearest city, ensuring that their products are of high quality and reasonably priced.

While the BCZ plays a role in the medical equipment procurement system, it does not appear to be the entity that purchases equipment, at least not systematically. One BCZ informant mentioned that their annual operational action plan for the health zone includes consideration of equipment, informed by the national equipment standards, but the informant did not mention the BCZ buying anything directly. A health center-based informant explained that sometimes the needed equipment is in stock at the BCZ, but it is not clear that the BCZ purchases equipment. Health zone staff explained that, within the government system, purchasing decisions are made at the national level, and that they do not have funding allocated for equipment. If the national level does not meet local needs, the BCZ may attempt alternative methods of acquiring needed equipment. One informant described appealing to the town hall, partners, and even individual benefactors.

We express our needs and when the national level wants to provide, it is they themselves who determine [what to provide]; […] for example in [the vaccination program], they say such refrigerator must go to such place even if we have an imminent need in another place, but as Kinshasa has already predetermined an institution where this material will go, we cannot change that. (Health zone – Katanga)

The National Plan for Health Development did not specify a role for provincial health offices related to equipment purchasing. However, DPS’s appear to purchase some expensive equipment for health facilities, particularly hospitals. One provincial-level official explained that they solicit input from end-users before purchasing, to ensure that what they buy is appropriate. However, these needs cannot always be fulfilled due to budgetary constraints.

They make orders based on missing equipment in their institutions. These are orders […] that remain archived because the orders are not fulfilled. (DPS)

#### International donor funds

Very often, medical equipment for public health facilities is purchased with donor funds of international organizations. In this case, health facilities or the organization purchasing equipment for them follow the procedures of the donor when ordering medical equipment. Private health facilities follow the procedures of the owners, although if they are a “designated” facility, they are theoretically subject to government requirements as well.

In general, partners sign contracts at the provincial level and operate through the BCZ. They are typically not supposed to coordinate with the health facilities directly; this includes providing them directly with medical equipment. Likewise, the health facility is not supposed to directly solicit partners for support. It is unclear whether this policy comes from the government or from partners, or both. There are some exceptions; in Haut Katanga, an informant explained that a partner had assisted their health center directly with procuring “enormously expensive” equipment. It also appears that hospitals feel freer to appeal directly to partners compared with health centers.

There was strong consensus among informants that partners do not give facilities funds for the purchase of equipment, but instead purchase it themselves. However, this sentiment was contradicted by an informant from a partner organization, who indicated that health facilities received financing with which they are expected to purchase medical equipment.

Some equipment… take, for example, blood pressure monitors get damaged, thermometers get damaged. These are equipment that are damaged. But the project does not purchase them. The project provides medicines and financing for operating costs. And from that, medical structures, health centers, hospitals, they will buy [equipment] considering their needs. (Implementing Partner)

A single health zone may have multiple partners providing equipment at the same time. Several BCZ informants discussed their expectations that partners would provide medical equipment and expressed disappointment that promised materials had not yet been provided by their current partner. It appears that, once a partner has committed to providing equipment, options for government procurement are typically not pursued, even in the face of delays from the partner.

Informants from partner organizations indicated that equipment is generally bought once for many health facilities, often based on their own evaluations of the needs in provinces they supported.

We only bought equipment once. […] But in the [project] proposals, the frequency is not determined, at least we can cover many health facilities. We did it once, but for many structures at once. (Implementing Partner)

#### Other sources of support

Informants reported that occasionally, small charity organizations such as church groups and visiting physicians donate equipment directly to health facilities. Politicians also sometimes visit facilities and bring them equipment. In these cases, the facility may accept the donation, and is supposed to report the donation to the BCZ.

Yes, the [health zone] office needs to know because the office has our backs. The person who brings us a gift may in the long run create problems for us. So, if the office does not know who is going to cover us and who is going to defend us. (Health center – Kasaï Oriental)

It does not appear that communities or community members purchase medical equipment for their health facilities, except for rare cases in which a patient breaks a piece of equipment, or a wealthy benefactor is asked to assist. However, communities can be quite involved in the procurement of medical equipment. CODESA members are supposed to assist in health centers’ annual planning and in making purchasing decisions. Community members may also be kept abreast of the status of equipment at the facility. One health center-based informant mentioned that when the facility receives new equipment, the community is invited to see it be unpacked and inventoried.

### Maintenance and depreciation

#### National guidelines

A national maintenance guide has been drafted at the national level but has not been disseminated at the time of this study. The guide includes information related to the preventive and curative maintenance of laboratory and surgical department equipment. For prevention, three columns are provided: designation, periodicity, and task to be performed. Table 2 outlines this configuration.

**Table 2:** Excerpt from national equipment preventive maintenance guide (draft) *(translated from French)*

Designation	Frequency	Task(s)
1. PH meter (Hydrogen potential)	Daily	Clean the window of light emitters and sensors with a small brush. Maintenance procedures of a Microplate Washer: 1. Check the delivered volume 2. Test the uniformity of the filling 3. Check the efficiency of the vacuum subsystem 4. Check the cleanliness of the dispensing and extraction needles Note: System maintenance must be performed by a qualified technician.
	Weekly	
	Monthly	
	Quarterly	Use the calibration plate and take readings with the same plate at 30-minute intervals. Compare the results. They must not present any differences. - Clean the plate holder drawer. -Check the alignment of each well with the light emitting and detecting systems.

For corrective maintenance, the document provides the designation, problems, probable causes, and solutions as shown in Table 3.

**Table 3:** Excerpt from national equipment corrective maintenance guide (draft) (*translated from French)*

Designation	Problems	Probable causes	Solutions
1. Pulse oximeter	The oximeter does not turn on	The battery is not in place The oximeter is not connected to the mains	Install batteries Check power supply Connect the device to the mains
	The alarm sound is not working	The alarm is not connected to the oximeter The probe is connected to an extension cable	Check that the probe is correctly connected with the oximeter Check that the extension cord is connected correctly with the oximeter
	“No finger” alarm	The sensor is not connected to the patient’s finger	Verify that the sensor is correctly connected to the patient’s finger

Since the standards are not published, each facility acts according to the approach that seems best to it. At the national level, responsible for regulating equipment standards, there are concerns raised regarding this practice.

At the level of the Provincial Health Divisions, informants appeared unaware of the national directives. Emphasis was placed on the manual that accompanies the equipment as a standard to follow.

Each equipment has its life sheet called the maintenance sheet. This sheet is drawn up as soon as the equipment is acquired, because when the equipment arrives there is first the after-sales service that must be provided. After this service, the equipment is provided, for example, to the service, and the latter begins to use it. It is then, since the day you installed it, you have a sheet on which you will record the date of acquisition, the date of installation and the date of commissioning. Then, from the date of commissioning, you can project (possibly 6 months later), do a check to see the performance of the equipment in relation to the constraints it has undergone (its operation), and therefore the first maintenance. (DPS)

#### Maintenance technicians

The number of technicians trained in equipment maintenance is not tracked at the national level. The equipment maintenance sector is new, with a single technical institute named ISTA (National Institute of Applied Techniques) serving the entire country. Responsibility for equipment maintenance falls to electricians, electronics engineers, or pharmacists, many of whom have only on the job training.

Maintenance workers in the DRC often learn through briefings rather than through a well-defined course.

Concerning having someone specifically trained for this repair, there are instead, I repeat myself, small briefings that we give on the repair of certain materials at the health center; in this case, I was briefed by the technician. For example, when we were provided refrigerators, there was the technician who had been sent by the partner, PATH, who briefed us on how to defrost the refrigerator, how to clean the solar system to prevent dust from covering the solar panels. (Health center – Tanganyika).

Theoretically, recruitment of maintenance workers is expected to follow the same principles as all other positions, either by competition (for directors) or by title (by presenting a diploma which makes them eligible for the position). In practice, interviews indicate that this is rarely the case.

Job descriptions are not defined, this is a great weakness. … In practice, recruitment decisions come from politicians or political authorities: we do not oppose them because they have the green pen … to say that the pen of the decision can even dismiss you from your post. (MoH)

#### Maintenance in practice

In health centers or hospitals, when someone notices an equipment breakdown, they report to their superiors to decide whether there is a need for external maintenance or not, or whether to remove the equipment from service. If the equipment was donated, the information passes from the health facility to the BCZ and from the BCZ to the DPS, but also from the BCZs to the partner who provided the equipment to find a technician who can repair it.

[The technician] will let us know so that we can check or replace other equipment. (Health center- Kasaï oriental)

There is an administrator in charge of assets who notices that the equipment is not working, they report it to the management, the Medical Director calls the committee together, we work on it, and we are going to call someone to fix it if it is still repairable […] it is the management that makes the decision. (Hospital - Haut Katanga)

So, each time, they are there also to check the […] materials that are dead, that no longer work. Then he reports. (Provincial Health Office)

For minor breakdowns of small equipment, the health facilities generally manage repairs. External maintenance assistance is required for major breakdowns on large equipment, by personnel who have training as an electrician, electronics engineer, or mechanic. If possible, technicians are recruited locally, but some cases require expertise from abroad.

There are items for which [we are] allowed to call [the maintenance worker], it is not all the equipment. As the refrigerator is something that the partner gave us for the PEV, it is the PEV that gave us that. For that I do not call maintenance, for that I inform the central office directly. It is the central office that will take care of it, for that it is not the institution’s responsibility. For those for which we call outside technicians, it is what we ourselves have purchased. But for those of the partners we do not touch, there we call the central office. (Health center – Kasaï Oriental)

Informants from partner organizations reported budgeting for and maintaining equipment. However, this may not always be possible due to budgetary constraints. When maintenance support could not be guaranteed, an informant indicated that they trained the health workers on preventative maintenance.

We procure at the start of the project and the project ensures maintenance. If, perhaps, there are batteries that are damaged while you are implementing the current project, […], if the warranty has expired, the project [replaces it]. There are always designated funds intended to replace [equipment] if there are breakages.

#### Depreciation

Informants from health zones were asked whether there was a schedule for the useful life of equipment (i.e., depreciation). In Kasaï Oriental, one health zone official said equipment was generally eligible for retirement after five years, although the informant did not specify what types of equipment were subject to that timeline. In contrast, another health zone representative in Kasaï reported that there was no schedule for equipment amortization. In Tanganyika, a health zone official replied that having an amortization calendar was a good suggestion that the health zone should implement and asked the interviewer for guidance on doing so.

The depreciation, we have not planned for that. But it is a really good idea, we’re going to capitalize on it, I don’t know if you can train us on it? (health zone office – Tanganyika)

### Summarization of results

The themes emerging from this study indicate a lack of data on equipment, a disconnect between the availability of data, resource mobilization power, and decision-making responsibility among the actors in the DRC health system. Decision makers at the provincial and national levels, who have access to resources, do not have the data necessary to make informed decisions, nor do they understand it to be their role to do so. Similarly, the individual institutions and health zone offices, which gather data on medical equipment, do not possess the resources to shore up gaps observed from national standards, although the responsibility for doing so falls at their level. Table 4 summarizes these findings.

**Table 4:** Resources, data availability and responsibility for equipment at all levels of the health system

Level	Resources available	Data available	Frequency	Responsibility for equipment*
National	Donors, National Budget	6–12 pieces of equipment	Yearly	Minimal
Provincial	Donors, Provincial Budget	6–12 pieces of equipment	Trimesterly	None
Health Zone	None	Full inventory	Monthly	Significant
Facility	Revenue, Community	Full inventory	Continuous	Significant

*Based on authors’ assessment

## Discussion

This study qualitatively assessed the design and operational functioning of the medical equipment system in the Democratic Republic of Congo, focusing on information systems, procurement processes, maintenance, and depreciation, and examined how roles and responsibilities, including those of government actors and development partners, influence equipment availability and functionality at health facilities.

The discordance between national standards and functional equipment in health facilities is likely not due to a lack of standards, resources, or data, but challenges in execution. The system would be strengthened if information on equipment needs was brought to those who have the resources and decision-making power for these needs to be addressed.

### National guidelines

The central government’s stated role in the medical equipment system is to develop and disseminate guidelines. The MoH has long defined the minimum package of medical equipment for health facilities. Recently, the MoH developed guidelines for equipment maintenance; these have yet to be disseminated. There do not appear to be guidelines on approved equipment specifications (i.e., make and model), which would promote uniformity of equipment among facilities and enable equipment to be more readily repaired, nor are there guidelines on amortization. The MoH could consider developing a comprehensive set of guidelines for medical equipment that includes equipment specifications and amortization.

There does not appear to be an audit mechanism to ensure that national guidelines are disseminated and followed, nor does there seem to be any official recourse if a facility is unable to achieve compliance with national standards under their own budget. The provincial health office could consider creating a province-level administrative position charged with disseminating standards to all who are expected to follow them and ensuring adherence to medical equipment guidelines.

### Equipment procurement

The system through which equipment needs are determined and fulfilled operates differently to what is described in government documents. While official documents indicate that the CDRs are responsible for equipment procurement, in practice the CDRs are seemingly uninvolved. There does not seem to be a clear understanding of who, within the MoH, is responsible for procuring equipment, or what the process is followed. The central government is largely uninvolved in medical equipment procurement due to the decentralized design of the DRC’s health system; however, the provincial health offices do not appear to play a significant role either. The absence of clear governmental or provincial support leaves a gap for health facilities to fill through relationships with donors and private sector procurement.

Occasionally, facilities request equipment from the health zone office, which may fill the request from its own stock, obtain it from a partner, or deny the request. In any case, there is no official process by which the facility receives a response. The Ministry of Public Health could consider institutionalizing a process for keeping facilities informed of the status of equipment requests. This could be done during facility supervision, during regular meetings, or through an electronic system.

When they have the means and the inventory is available, health facilities may purchase needed equipment from the private market. While this can be a financial hardship for some facilities, several of the facilities visited for this study reported engaging in regular planning and budgeting exercises that include medical equipment procurement. Facilities in these zones appeared to have a better understanding of the equipment procurement process. Additionally, these facilities used their equipment data to inform prospective budgets and management plans. One of these facilities was also in a health zone supported by the World Bank’s Performance Based Financing program, which also promotes this type of planning and provides subsidies for equipment procurement. The MoH and partners could consider expanding this process of regular planning and budgeting, including medical equipment, to all health facilities. They might also explore whether and in what cases subsidies may be necessary for facilities to achieve the minimum package of equipment.

**Fig. 2:**
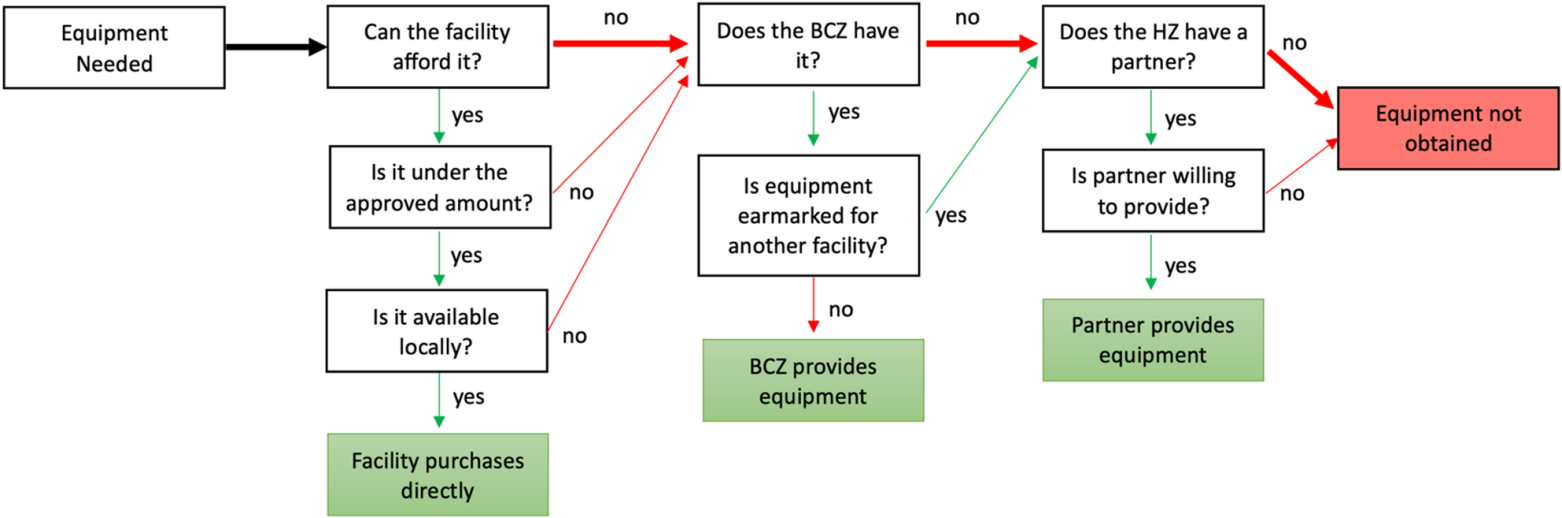
Procurement processes for health facilities

Overall, the medical equipment procurement system appears ill-defined, both on paper and in practice. The MoH could consider a full review and revision of the system so that processes and roles are well-documented and feasible under current conditions. This should include all levels of the government public health system (MoH, CDRs, provincial health office, health zone office, health facilities, CODESAs) with an option for donor/partner involvement. A diagram of the current procurement process, as informed by this study’s findings, is provided in Fig. 2, with the most common pathways highlighted in bold.

### Equipment maintenance

The communication channel for reporting inoperable equipment appears to be functioning as designed, with facilities regularly reporting the numbers of non-functional pieces of equipment through the SNIS. The country has made significant investment in its routine health information system, and facilities are reporting the required equipment data. While the equipment data forms are confusing and of limited scope, and the data that they produce is inadequate for decision making, the fact that the reporting system functions presents an opportunity. In the short term, rather than tracking the “number of days non-functional” by equipment type, facilities could report the number of functional pieces of each equipment type by month.

In the longer-term, the SNIS platform is not designed to be an equipment inventory management system and in fact, an ad hoc, paper-based methods for tracking equipment have emerged to fill that gap. Investment in a dedicated, electronic system for inventory tracking, maintenance, projection, and budgeting would produce the data necessary for proactively managing medical equipment, both within individual health centers and hospitals and at higher levels of the health system (health zones and provinces). The World Health Organization has published technical guidance on the design of such a system (World Health Organization, 2011). An electronic equipment procurement system, like the one used for medicines, could also be considered. At minimum, a dedicated database for medical device shortages and maintenance, akin to the Medical Device Shortage List [5], would allow actors other than the health facilities themselves to respond to the most urgent equipment needs to protect population health.

In general, there does not appear to be a process by which facilities request and receive maintenance on broken medical equipment. While national guidelines state that each facility should employ a maintenance technician, in practice, facility-based technicians are almost universally absent. This may be, in part, a function of supply, as there are a limited number of training programs for medical equipment maintenance technicians in the country. In the absence of such a system, facilities try to repair equipment themselves, or hire local technicians who may not have the necessary expertise. The inadequate number of repair technicians in the DRC deserves attention by the MoH. As a stopgap measure, provincial health offices could consider employing a small number of technicians who could be incorporated in routine supervision and service high-priority equipment throughout the province. National specifications for equipment procurement could also consider the availability of spare parts and trained technicians. Furthermore, the government and its partners could seek ways to encourage the growth of an indigenous maintenance and repair industry.

### The role of donors and partners

Official documents do not mention the role of partners or foreign donors in equipment procurement, despite their major roles. While the objective of large integrated health programs is usually health system strengthening, in practice these programs often provide at least some medical equipment as well. Those interviewed for this study explained that the health zone office is supposed to act as an intermediary between facilities and partners, meaning that facilities are not able to directly request equipment from a partner. Further, the process by which donors and partners make decisions on what equipment is provided and where it is distributed, and whether and how those decisions should be coordinated with the government, does not appear to be documented.

Donors will likely continue to operate in the DRC in the long-term. The government could improve the medical equipment system by working with partners to clearly delineate roles regarding equipment procurement. If donors are expected to be solely responsible for equipment, this should be clarified, and guidelines with specifications on the type of equipment expected at each level of the health system need to be made available to donors. If donors are not willing to take on that function, the government should make other arrangements, so facilities are not left unsupported.

In the current development climate, donors tend to be more interested in investing in system strengthening than in buying equipment. However, given the DRC’s weak medical equipment system, programs will likely need to support the procurement and repair of equipment in conjunction with their other activities. To strengthen the system, the government could request funding for a comprehensive medical equipment system, including training in equipment systems management and maintenance. This would have sustained impact on health services in the areas directly supported by the donor, and spillover effects to the rest of the country.

In our interviews, government officials could not articulate a plan for maintaining national equipment standards in the absence of external funding and procurement, and at least one health zone official stated that the province does not budget for medical equipment but cedes that responsibility to the donor. Informants in Kinshasa described “orphan health zones”, or zones with no donor support, and indicated that there is no functioning equipment procurement system in such zones. Establishing such a system in the absence of donor support would likely improve health services in those places. It would create opportunities to pilot and evaluate the effectiveness of different system designs that could be scaled elsewhere, ultimately moving the DRC’s health system closer to donor independence.

### Limitations

While efforts were made to conduct a comprehensive assessment of government policies and procedures related to the medical equipment system, the decentralized nature of the DRC’s health system means that some relevant documents may have been missed. Health facility interviews were performed in provinces receiving substantial donor support, which may limit their generalizability. Furthermore, due to logistical and financial constraints, transcript validation and member checking could not be performed among respondents.

This study did not explore in detail private sector equipment manufacturers, distributors, or maintenance technicians beyond those referenced in national documents or referred to by respondents.

## Conclusions

This study identifies several weaknesses in the medical equipment system in the DRC, ultimately impacting the availability and functioning of essential medical equipment in public health facilities. The system’s inefficiencies contribute to facilities lacking the minimum required equipment and experiencing challenges in equipment maintenance. Clear guidelines on equipment specifications, maintenance, and procurement are lacking, and there is a lack of coordination and understanding regarding the responsibility for equipment procurement within the government system.

Our findings highlight the need for a comprehensive review and reform of the medical equipment system, including the involvement of all relevant stakeholders. Further, there is a need to clarify the roles and responsibilities of partners and donors in equipment procurement and make strides toward a functioning system in the absence of donor support. The government should also consider investing in a dedicated equipment inventory management system and training programs for maintenance technicians. Likewise, our findings reinforce the importance of prioritizing medical equipment, technical and financial support by international partners for LMICs. By addressing these issues, the DRC can sustainably strengthen its medical equipment system and improve health service delivery throughout the country.

## Supplementary Information

Below is the link to the electronic supplementary material.

Supplementary Material 1

Supplementary Material 2

Supplementary Material 3

Supplementary Material 4

Supplementary Material 5

## Data Availability

The datasets generated and/or analyzed during the current study are not publicly available due to the confidential nature of the interview transcripts but are available from the corresponding author on reasonable request.

## Abbreviations

AG: Administrator-Manager | Administrateur-Gestionnaire
ASSP: Accès aux Soins de Santé Primaires
BCZ: Health Zone Central Office | Bureau Central de la Zone de Santé
CDR: Regional Distribution Center | Centre de Distribution Régionale
CODI: Hospital Management Committee | Comité de Direction
CODESA: Health Area Development Committee | Comité de Développement de l’Aire de Santé
CS: Health Center | Centre de Santé
D4I: Data for Impact
DHIS2: District Health Information System 2
DPS: Provincial Health Division | Division Provinciale de la Santé
DRC: Democratic Republic of Congo
ECZ: Health Zone Management Team | Équipe Cadre de la Zone de Santé
HGR: General Reference Hospital | Hôpital Général de Référence
IHP: Integrated Health Program
INGO: International Non-Governmental Organization
ISTA: National Institute of Applied Techniques | Institut National des Techniques Appliquées
LMICs: Low- and Middle-Income Countries
MCZ: Health Zone Chief Physician | Médecin Chef de Zone
MoH: Ministry of Public Health | Ministère de la Santé Publique
PEV: Expanded Programme on Immunization | Programme Élargi de Vaccination
PNAME: National Essential Medicines Supply Program | Programme National d’Approvisionnement en Médicaments Essentiels
SNIS: National Health Information System | Système National d’Information Sanitaire
USAID: United States Agency for International Development
WHO: World Health Organization
