# Supplementary material for: Assessment of the medical equipment supply chain in the Democratic Republic of Congo: a qualitative methods study
Source: BMC Health Serv Res. 2026 Feb 5;26:340. doi: 10.1186/s12913-026-14131-y (PMC12973841; doi:10.1186/s12913-026-14131-y)
Supplement: Supplementary file 1 — Supplementary Material 1 [file 12913_2026_14131_MOESM1_ESM.docx]

# Appendix A. Documents reviewed

1. Loi n° 18/035 du 13 décembre 2018 fixant les principes fondamentaux relatifs à l'organisation de la Santé publique
2. Loi relative aux marches publics (avril 2010)
3. Décret n° 010/34 du 28 décembre 2010 fixant les seuils de passation, de contrôle et d'approbation des marchés publics
4. Décret n° 10/22 du 02/06/2010 portant manuel de procédures de la loi relative aux marches publics
5. Décret n° 10/21 du 02 juin 2010 portant création, organisation et fonctionnement de l’autorité de régulation des marches publics, en sigle « ARMP »
6. Décret n°10/22 du 02 juin 2010 portant manuel de procédures de la loi relative aux marches publics
7. Rapport d’évaluation du Plan stratégique de Sécurisation des Produits de Santé de la Reproduction 2008-2012 de la République Démocratique du Congo
8. Manuel de procédures de passations de marches applicables aux financements sous gestion de la CAGF (Tome 7)
9. Procédures opérationnelles standards de la CAMESKIN
10. Rapport mensuel Division Provinciale de la Santé
11. Rapport mensuel du BCZ
12. Rapport mensuel du centre de santé
13. Rapport mensuel de l’hôpital
