# Supplementary material for: Assessment of the medical equipment supply chain in the Democratic Republic of Congo: a qualitative methods study
Source: BMC Health Serv Res. 2026 Feb 5;26:340. doi: 10.1186/s12913-026-14131-y (PMC12973841; doi:10.1186/s12913-026-14131-y)
Supplement: Supplementary file 2 — Supplementary Material 2 [file 12913_2026_14131_MOESM2_ESM.docx]

# Appendix B. SNIS data entry forms for medical equipment: Health center

**Original French**

4.3 Matériel et Equipment : Jrs de non-Fonctionnalité (Partie 1)

|  | Valeur |
| --- | --- |
| Électricité |  |
| Frigo |  |
| Microscope |  |
| Glucomètre |  |
| Spectrophotomètre |  |
| Centrifugeuse |  |

**English translation**

4.3 Material and Equipment: Days of non-functionality (Part 1)

|  | Value |
| --- | --- |
| Electricity |  |
| Refrigerator |  |
| Microscope |  |
| Glucometer |  |
| Spectrophotometer |  |
| Centrifuge |  |
