# Supplementary material for: Assessment of the medical equipment supply chain in the Democratic Republic of Congo: a qualitative methods study
Source: BMC Health Serv Res. 2026 Feb 5;26:340. doi: 10.1186/s12913-026-14131-y (PMC12973841; doi:10.1186/s12913-026-14131-y)
Supplement: Supplementary file 3 — Supplementary Material 3 [file 12913_2026_14131_MOESM3_ESM.docx]

# Appendix C. SNIS data entry forms for medical equipment: Hospital

**Original French**

4.12 Matériel et Equipment

|  |  | Nombre Jrs Non Fonctionnelle |
| --- | --- | --- |
| Ordinateur |  |  |
| Photocopieuse |  |  |
| Moto |  |  |
| Véhicule |  |  |
| Internet |  |  |
| Incubateur |  |  |
| Chaine pour électrophorèse |  |  |
| Couveuse |  |  |
| Appareil de réanimation |  |  |
| Échographe |  |  |
| Radiographie |  |  |
| Électrocardiogramme |  |  |

**English translation**

4.12 Material and Equipment

|  |  | Number of Days Non-functional |
| --- | --- | --- |
| Computer |  |  |
| Photocopier |  |  |
| Motorcycle |  |  |
| Vehicle |  |  |
| Internet |  |  |
| Incubator (pre-term) |  |  |
| Electrophoresis chain |  |  |
| Incubator (full-term) |  |  |
| Resuscitation device |  |  |
| Ultrasound |  |  |
| X-ray |  |  |
| Electrocardiogram |  |  |
