# Supplementary material for: Assessment of the medical equipment supply chain in the Democratic Republic of Congo: a qualitative methods study
Source: BMC Health Serv Res. 2026 Feb 5;26:340. doi: 10.1186/s12913-026-14131-y (PMC12973841; doi:10.1186/s12913-026-14131-y)
Supplement: Supplementary file 4 — Supplementary Material 4 [file 12913_2026_14131_MOESM4_ESM.docx]

**Key informant interview guide**

**Channel 1: Communicating that equipment is non-functional to the SNIS**

1. Do the items on the Canvas forms align with minimum standards for equipment that should be present in health centers/hospitals?
2. Is it accurate that the health centers and hospitals send the paper forms to the BCZ, who enters it into the SNIS?
3. Who is supposed to review the equipment data?
4. Who is supposed to act on non-functional equipment?
5. The national staffing standards say that each facility should have a “maintenance tech.” What is the job description for that role? What is their training/background and how are they recruited and paid?

**Channel 2: Reporting that new equipment is needed to the CDR**

1. What equipment is listed in the order form? Does it align with minimum standards for equipment that should be present in health centers/hospitals?
2. How do facilities determine what to order and when? Are facility directors aware of the minimum standards for equipment?
3. Who pays for equipment? Is it the facility, the BCZ, someone else?
4. Who would make the decision to purchase from a private supplier rather than the CDR?
5. Who sets the budget for equipment procurement and repair? How is it determined? Are facilities made aware of the equipment budget?
6. If requested equipment cannot be obtained, how is that message communicated back to the facility?
7. Is there an official maintenance or retirement schedule for equipment?
8. Do CDR’s supply parts or just equipment?
9. What information is tracked in the CDR’s database?

**Partner support**

1. Can partner-supported facilities order equipment from the CDR or are they expected to procure through the partner?
2. What partners have supported this area? What do/did they provide in terms of medical equipment?
