# Supplementary material for: Assessment of the medical equipment supply chain in the Democratic Republic of Congo: a qualitative methods study
Source: BMC Health Serv Res. 2026 Feb 5;26:340. doi: 10.1186/s12913-026-14131-y (PMC12973841; doi:10.1186/s12913-026-14131-y)
Supplement: Supplementary file 5 — Supplementary Material 5 [file 12913_2026_14131_MOESM5_ESM.docx]

**In-depth interview guides**

Facility-level data generation

1. What data is collected now, how, at what frequency, and by whom?
   1. Health center inventory- counts, age, condition
   2. Trainings on equipment use and repair
2. Where is the data stored?
3. Are data governance policies followed?
4. Are/how are data cleaned and processed?

Data access

1. Who has access to the data?
2. How do they access the data, and how reliable is their access? (internet, travel, etc.)
3. How is the data displayed: dashboards? raw form?

Data use

1. How often is the data accessed and by whom?
2. Are there set standards for the equipment that a facility should have, and are these referenced in dashboards or decision-making? Are individuals at each level aware of these standards?
3. Is there an official maintenance or depreciation schedule, and is it used?
4. Does the facility have a maintenance tech? If yes, who are they and what can and can’t they do?
5. What data is used in budgeting and planning?
6. Can/do decision-makers request ad hoc analyses?

Communication

1. How and to whom do facilities communicate that a piece of equipment is missing or broken, or that they need something new?
2. What is the role of a partner organization that supports a facility?
   1. Do they purchase equipment for the facility or provide funding for the purchase of equipment?
   2. Are equipment needs communicated to the partner and if so, how?
3. To what extent are facilities free/able to purchase equipment directly?
4. How do facilities communicate that they have received a direct donation?
5. How are decisions/statuses re: equipment communicated back to the facilities?
6. How are decisions/statuses re: equipment communicated to communities?

Health center/hospital equipment case study
*Data collectors will be provided with a medical equipment report card for the facility based on data from D4I’s midline survey. They will discuss pre-selected pieces of equipment with the respondent, documenting the process by which it was obtained and the schedule for its maintenance and replacement. They will also discuss the equipment that the facility is missing and explore the reasons why the facility is not adequately equipped.*

**Health center/hospital equipment case study guide**

| **Equipment** | **# Present** | **# Operational** |
| --- | --- | --- |
| Stethoscope | 1 | 1 |
| Thermometer | 2 | 1 |
| Blood pressure monitor | 1 | 1 |
| Adult scale | 0 | 0 |
| Infant scale | 0 | 0 |
| Light source (spotlight) | 1 | 0 |
| Autoclave | 0 | 0 |

***Select a piece of equipment that was present and operational at the time of the midline survey. Ask the respondent the following questions:***

1. Is the piece of equipment still present?
   1. If no, why?
2. Is it still operational?
   1. If no, why?
   2. If yes, has it been repaired or serviced since the survey? By whom? Paid for by whom?
3. How did the facility acquire this piece of equipment?
   1. When was it acquired?
   2. From where was it acquired?
   3. Who paid for it?
   4. Was it acquired new or used?
4. How much longer do you estimate that this piece of equipment will last?
5. If this piece of equipment were to break today, how long do you think it would take to get it repaired?
6. If this piece of equipment were to be completely damaged today, how long do you think it would take to replace it?

***Select a piece of equipment that was present but not operational at the time of the midline survey. Ask the respondent the following questions:***

1. Is the piece of equipment still present?
   1. If no, why?
2. Is it now operational?
   1. If no, why?
   2. If yes, who repaired or serviced it? Who paid for the repair?
3. How did the facility acquire this piece of equipment?
   1. When was it acquired?
   2. From where was it acquired?
   3. Who paid for it?
   4. Was it acquired new or used?

***Select a type of equipment that was not present at the time of the midline survey. Ask the respondent the following questions:***

1. Have you acquired this piece of equipment?
   1. If no, have you requested it?
   2. From whom?
   3. Why has it not been obtained?
2. If yes, how did the facility acquire this piece of equipment?
   1. When was it acquired?
   2. From where was it acquired?
   3. Who paid for it?
   4. Was it acquired new or used?
